# Enhancing Musculoskeletal Competency: A Quality Improvement Project

**DOI:** 10.51894/001c.164408

**Published:** 2026-08-01

**Authors:** Maan Faraj, Fatin Sahhar

**Affiliations:** 1 Detroit Medical Center - Sinai Grace Hospital

## 51

### Background

Musculoskeletal (MSK) complaints are among the most common reasons for visits in Family Medicine. However, variability in MSK examination and procedural skills among trainees can lead to inconsistent evaluation, delayed diagnosis, and suboptimal management. Within our Family Medicine residency program, a gap was identified in the consistent performance and documentation of MSK examinations during clinical encounters, representing an opportunity to improve care processes.

### Objectives

The objective of this Quality Improvement (QI) project was to increase the proportion of clinical encounters in which Family Medicine residents appropriately performed and documented MSK examinations and procedures. Over a six-month period, the project aimed to improve appropriate MSK examination and procedural performance from a baseline of 36% to at least 50%.

### Methods

This project was conducted as a QI initiative using a Plan–Do–Study–Act (PDSA) framework within a Family Medicine residency program. Baseline performance was assessed using standardized pre-intervention evaluations measuring residents’ ability to appropriately perform MSK examinations and procedural decision-making relevant to clinical practice. The intervention consisted of a structured curriculum of alternating didactic lectures and hands-on MSK workshops focused on common MSK conditions encountered in Family Medicine. Sessions emphasized standardized examination techniques and appropriate procedural skills and were facilitated by faculty and senior residents. Outcomes were measured using matched pre- and post-intervention assessments, and aggregate data were analyzed to assess changes in performance.

### Results

Following implementation of the intervention, the proportion of residents appropriately performing and documenting MSK examinations and procedures increased from 36% at baseline to 71%, exceeding the predefined improvement goal. Improvement was observed across multiple MSK topics and was more pronounced following hands-on workshop sessions.

### Conclusions

This QI initiative demonstrated that a structured, skills-based MSK curriculum was associated with meaningful improvement in clinical practice behaviors among Family Medicine residents. Future QI cycles will focus on sustaining gains through standardized documentation tools, increased direct observation and feedback, and ongoing skills reinforcement to further improve MSK care delivery

